# 2391. Evaluation and Adjudication of Case Reports of Myocarditis After mRNA-1273 Vaccination

**DOI:** 10.1093/ofid/ofad500.2011

**Published:** 2023-11-27

**Authors:** Veronica Urdaneta, Priyadarshani Dharia, Oketoun Akangbe, Samantha St Laurent, Daina Esposito, Magalie Emile-Backer, Walter Straus

**Affiliations:** Moderna, Inc., Cambridge, Massachusetts; Moderna, Inc., Cambridge, Massachusetts; Moderna, Inc., Cambridge, Massachusetts; Moderna, Inc., Cambridge, Massachusetts; Moderna, Inc., Cambridge, Massachusetts; Moderna, Inc., Cambridge, Massachusetts; Moderna, Inc., Cambridge, Massachusetts

## Abstract

**Background:**

Myocarditis and/or pericarditis have been identified as very rare adverse events (AEs) associated with administration of COVID-19 vaccines, particularly mRNA vaccines. Cases were disproportionately reported in young men aged 18-24 years, within a few days after vaccination, mostly following a second dose. This analysis describes findings of the evaluation and adjudication of myocarditis/pericarditis AE reports (18 Dec 2020 – 17 Dec 2022) in the Moderna Global Safety Database (GSDB) following mRNA-1273 vaccination.

**Methods:**

Two different case definitions were used to characterize level of diagnostic certainty and identify strength of evidence supporting a diagnosis of myocarditis/pericarditis: the Brighton Collaboration (BC) Case Definition (1-5 categories) and the Centers for Disease Control and Prevention (CDC) definition (probable/confirmed). Causality assessment was conducted using the standard WHO-UMC criteria. Based on epidemiological criteria, analysis was concentrated on reported cases involving individuals aged <40 years, including < 18 years, after >2 doses of mRNA-1273 and bivalent vaccines, regardless of the time-to-onset of AEs after vaccination.

**Results:**

There were 6702 myocarditis/pericarditis cases after an estimated 773 million doses of mRNA-1273 (monovalent) or bivalent vaccine administered during the analysis period. There were no significant temporal changes in the demographic distribution of reported cases; 66.2% of these cases occurred in men aged 18-39 years (Figure 1) and after the second dose (28.3%). In individuals < 40 years of age, according to the BC, 38.1% of the cases were confirmed/probable; according to CDC definition 37% of the cases were confirmed/probable. WHO causality assessment indicated that 39.8% of the cases were probable/possible.

Figure 1

**Figure:**
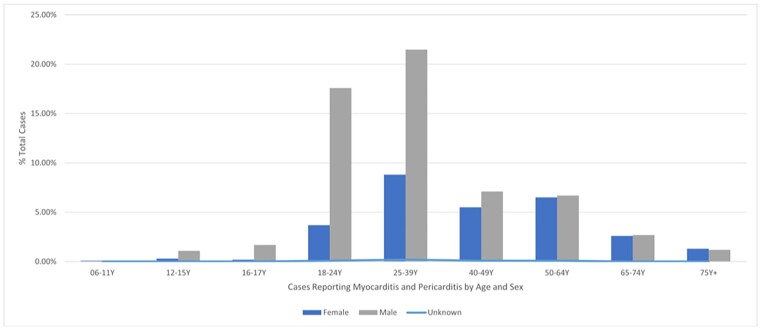

Percentage of mRNA-1273, mRNA-1273.214 (Original/BA.1) and mRNA-1273.222 (Original/ BA.4/5) Reported Cases of Myocarditis and Pericarditis by Age and Sex (18 December 2022 to 17 December 2022)

**Conclusion:**

Adjudication of myocarditis/pericarditis cases following mRNA-1273 indicated that where information was available, most reports were Probable Cases, for both BC and CDC case definitions. According to WHO-UMC causality assessment most cases were considered possibly related to mRNA-1273.

**Disclosures:**

**Veronica Urdaneta, MD, MPH**, Moderna, Inc.: Salary|Moderna, Inc.: Stocks/Bonds **Priyadarshani Dharia, PhD, MD, MPH**, Moderna, Inc.: Salary|Moderna, Inc.: Stocks/Bonds **Oketoun Akangbe, PharmD**, Moderna, Inc.: Salary|Moderna, Inc.: Stocks/Bonds **Samantha St Laurent, MPH**, Moderna, Inc.: Salary|Moderna, Inc.: Stocks/Bonds **Daina Esposito, PhD, MPH**, Moderna, Inc.: Salary|Moderna, Inc.: Stocks/Bonds **Magalie Emile-Backer, PharmD, CCRP**, Moderna, Inc.: Salary|Moderna, Inc.: Stocks/Bonds **Walter Straus, MD, MPH**, Moderna, Inc.: Salary|Moderna, Inc.: Stocks/Bonds

